# Study on the progression types of cancer in patients with breast cancer undergoing eribulin chemotherapy and tumor microenvironment

**DOI:** 10.1186/s12967-018-1443-5

**Published:** 2018-03-09

**Authors:** Shinichiro Kashiwagi, Gen Tsujio, Yuka Asano, Wataru Goto, Koji Takada, Katsuyuki Takahashi, Tamami Morisaki, Hisakazu Fujita, Tsutomu Takashima, Shuhei Tomita, Masahiko Ohsawa, Kosei Hirakawa, Masaichi Ohira

**Affiliations:** 1 Department of Surgical Oncology, Osaka City University Graduate School of Medicine, 1-4-3 Asahi-machi, Abeno-ku, Osaka, 545-8585 Japan; 2 Department of Pharmacology, Osaka City University Graduate School of Medicine, 1-4-3 Asahi-machi, Abeno-ku, Osaka, 545-8585 Japan; 3 Department of Scientific and Linguistic Fundamentals of Nursing, Osaka City University Graduate School of Nursing, 1-5-17 Asahi-machi, Abeno-ku, Osaka, 545-0051 Japan; 4 Department of Diagnostic Pathology, Osaka City University Graduate School of Medicine, 1-4-3 Asahi-machi, Abeno-ku, Osaka, 545-8585 Japan

**Keywords:** Tumor-infiltrating lymphocytes, Progressive disease, Breast cancer, Eribulin, Tumor microenvironment

## Abstract

**Background:**

Recently, the concepts of progression due to pre-existing lesions (PPL) and progression due to new metastasis (PNM) have been proposed to differentiate the progression types of treatment-resistant cancers. Previously, the differences between these two progression types did not affect the determination of treatment strategies since both PPL and PNM are classified as progressive disease based on the response evaluation criteria in solid tumors (RECIST) diagnostic criteria. On the other hand, tumor infiltrating lymphocytes (TILs) are effective when used as indicators for monitoring the immune tumor microenvironment (iTME) in the cancer host, and TILs play an important role as biomarkers in predicting prognosis and therapeutic effects. This study focused on the progression types of cancer in patients undergoing eribulin chemotherapy. In addition, the iTME in individuals with PPL and PNM was evaluated using TILs as a marker.

**Methods:**

Of the 52 patients with locally advanced or metastatic breast cancer who underwent chemotherapy with eribulin, 40 remained in the study, and 12 patients were dropout cases. The antitumor effect was evaluated based on the RECIST criteria using version 1.1. TILs were defined as the infiltrating lymphocytes within tumor stroma and were expressed in proportion to the field investigated. In PPL cases, the high-TIL group was considered as type I and the low-TIL group was classified as type II. In PNM cases, the high-TIL group was considered as type III and the low-TIL group was classified as type IV.

**Results:**

In 19 cases, individuals with type I progression had significantly longer progression free survival and overall survival (OS) compared to those with type III progression (p = 0.040, p < 0.001, log-rank). Individuals with type I progression had significantly prolonged survival post progression compared to those with type II progression (p = 0.048, log-rank). A multivariate analysis that validate the effect of OS showed that these were independent factors of good prognosis (p = 0.003; hazard ratio [HR] = 0.065) (p = 0.006; HR = 0.105).

**Conclusions:**

The effects of eribulin chemotherapy suggested that patients with progressive-type breast cancer that proliferates in a good iTME may have a good prognosis.

**Electronic supplementary material:**

The online version of this article (10.1186/s12967-018-1443-5) contains supplementary material, which is available to authorized users.

## Background

The evaluation of the therapeutic effects of chemotherapy for solid tumors based on the response evaluation criteria in solid tumors (RECIST) plays an important role in determining treatment strategies both in clinical trials and practice [1]. Recently, the concepts of progression due to pre-existing lesions (PPL) and progression due to new metastasis (PNM) have been proposed to differentiate the progression types of treatment-resistant cancers [2, 3]. Previously, the differences between these two progression types did not affect the determination of treatment strategies since both PPL and PNM are classified as progressive disease (PD) based on the RECIST diagnostic criteria. However, to date, it is known that PPL does not involve metastasis but only invasion to the peripheral tissues. In contrast, PNM involves both invasion into the peripheral tissues and metastasis to other organs.

Tumor infiltrating lymphocytes (TILs) are effective when used as indicators for monitoring the immune tumor microenvironment (iTME) in the cancer host, and TILs play an important role as biomarkers in predicting prognosis and therapeutic effects [4–6]. The tumor microenvironment (TME) influences tumor survival and growth, infiltration, and metastasis and has been a topic of interest because of its effect on tumor cells. In addition, it has also been considered as a new therapeutic target [7, 8].

Eribulin mesylate (eribulin), as a tubulin inhibitor, has cytocidal effects, and it has unique pharmacological properties that were proven to modulate the TME [9, 10]. In a phase III clinical trial on patients with locally advanced or metastatic breast cancer (MBC), eribulin significantly prolonged the overall survival (OS) of patients with the therapeutic effects of TME [11, 12]. Furthermore, this survival curve showed a characteristic pattern called the delayed separation curve in immune checkpoint inhibitor therapy and immunotherapy, thus emphasizing the effects of eribulin on the iTME.

Therefore, this study focused on the progression types of cancer in patients undergoing eribulin chemotherapy. The purpose of this study is to investigate factors contributing to the extension of OS of eribulin chemotherapy. In addition, the iTME in individuals with PPL and PNM was evaluated using TILs as a marker.

## Methods

### Patient background

**Fig. 1:**
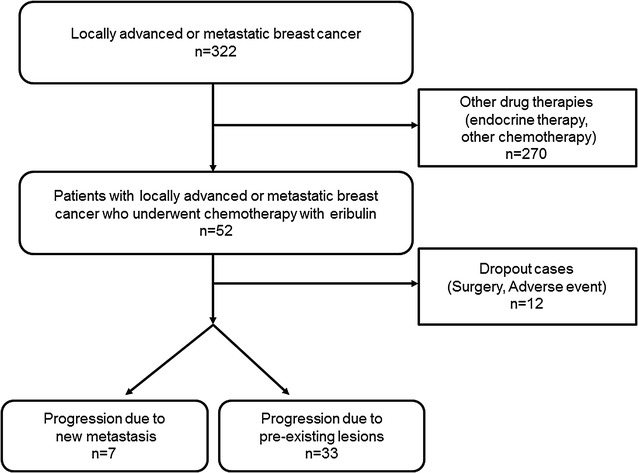
Consort diagram. A total of 322 patients with MBC underwent cancer treatment at Osaka City University Hospital from August 2000 to June 2013. In the present study, only 40 patients were included, and 270 patients with other drug therapies and 12 patients with dropout cases due to surgery or adverse events were excluded

A total of 322 patients with MBC underwent cancer treatment at Osaka City University Hospital from August 2000 to June 2013. In the present study, only 40 patients were included, and 270 patients with other drug therapies (endocrine therapy and other chemotherapeutic regimens) and 12 patients with dropout cases due to surgery or adverse events were excluded (Fig. 1). This data set was also used in previous studies [13, 14].

The median follow-up time was 431 days (range 50–650 days). The objective response rate (ORR), OS, progression free survival (PFS), and survival post progression (SPP) were obtained based on the efficacy of this regimen. The ORR was evaluated by adding the complete response (CR) and partial response (PR). The OS was defined as the period from the start date of treatment to death (daily). The PFS was defined as the period from the start date of treatment to either the earlier date of death or confirmation of PD (daily). The SPP was evaluated daily and defined as the period from the start date of the treatment after PD with eribulin chemotherapy to death. The antitumor effect was evaluated based on the RECIST criteria using version 1.1 [1].

Based on the chemotherapy regimen, which is one course of treatment for 21 days, eribulin (1.4 mg/m^2^) was intravenously administered on days 1 and 8 [11, 12]. This protocol was repeatedly used until PD was evaluated or it was discontinued due to severe adverse events.

The morphology of the tumor (type of histological tissue and nucleus grade) was identified using hematoxylin and eosin (H.E.) staining. Moreover, the expression of estrogen receptor (ER), progesterone receptor (PgR), human epidermal growth factor receptor 2 (HER2), and Ki67 were immunohistologically evaluated.

### Histopathological evaluation

Upon breast cancer diagnosis, TILs were evaluated by measuring the percentage of area occupied by the lymphocytes on the H.E.-stained tumor section using biopsy specimens [15]. The area of stromal TILs surrounding the stained cancer cells was quantitatively measured in each field of view (400×) [13, 16]. The area of the stroma with lymphoplasmacytic infiltration around the invasive tumor cell nests was > 50, > 10–50, ≤ 10%, or absent, and the corresponding scores assigned were 3, 2, 1, or 0, respectively (Additional file 1: Figure S1). TILs were classified as high (score of 2 or higher) and low (score of 1 and 0). TILs were histopathologically evaluated by two professional breast cancer pathologists.

### Classification based on progression type and evaluation of TILs

According to the RECIST guideline, PPL is the 20% increase in the sum of the diameters of the target lesions, and taking into consideration the small relative sum obtained in the study, an absolute increase of at least 5 mm was observed. PNM was defined as a lesion identified on a follow-up study in an anatomical location that was not assessed at baseline and is considered as a new lesion that can indicate disease progression [2, 3]. When PPL and PNM were observed at the same time during evaluation, PNM was considered. In PPL cases, the high-TIL group was considered as type I and the low-TIL group was classified as type II. In PNM cases, the high-TIL group was considered as type III and the low-TIL group was classified as type IV.

### Statistical analysis

A statistical analysis was performed using the SPSS^®^ version 19.0 statistical software (IBM, Armonk, NY, USA). The association between TILs and other clinicopathologic parameters was analyzed via the Chi square test (or Fisher’s exact test when necessary). The association with PFS, OS, and SPP was analyzed via the Kaplan–Meier plot and log-rank test. Univariate and multivariate hazard ratios (HR) were computed for the study parameters with 95% confidence intervals (CIs) using a Cox proportional hazards model, and a backward stepwise method was used for variable selection in multivariate analyses. A *p*-value less than 0.05 was considered statistically significant.

### Ethics statement

The study design involved a retrospective chart review. An informed consent was obtained from all patients according to the protocol approved by the Ethics Committee of Osaka City University (#926). This research is in accordance with the 2013 Declaration of Helsinki.

## Results

### Differences in progression types and prognostic analysis

**Fig. 2:**
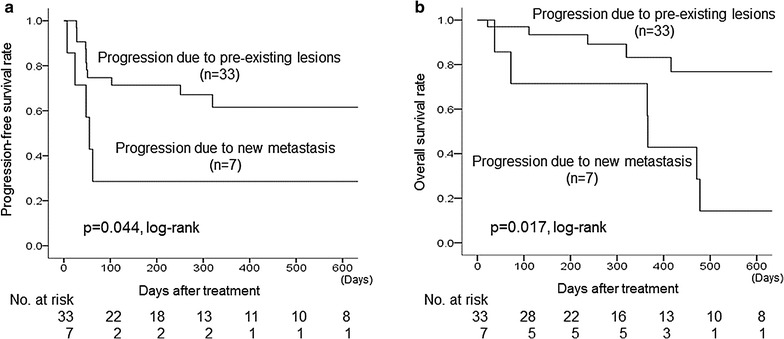
Differences in progression types and prognostic analysis. The 33 PPL group had a significantly longer PFS (p = 0.044, log-rank) (**a**) and OS (p = 0.017, log-rank) (**b**) compared to the 7 PNM group

Of the 52 patients with MBC who underwent chemotherapy with eribulin, 40 remained in the study, and 12 patients were excluded. Of which, 7 PNM cases (17.5%) and 33 PPL cases (72.5%) were observed. The PPL group had a significantly longer PFS (p = 0.044, log-rank) and OS (p = 0.017, log-rank) compared to the PNM group (Fig. 2).

### TIL expression and differences in progression types

Of the 40 participants, 23 (57.5%) were included in the high-TIL group, and 17 (42.5%) were classified in the low-TIL group. Of the 7 individuals in the PNM group, 4 were classified in the high-TIL group (57.1%), and 3 were included in the low-TIL group (42.9%). Of the 33 participants in the PPL group, 19 were classified in the high-TIL group (57.6%) and 14 were included in the low-TIL group (42.4%). There was no significant difference between clinicopathological parameter and TILs when group was divided by difference of TILs expression (Table 1).

**Table 1:** Correlations between tumor-infiltrating lymphocytes and clinicopathological parameters in 40 patients with eribulin chemotherapy for locally advanced or metastatic breast cancer

Parameters	All breast cancer (n = 40)		p-value	Progression due to new metastasis (n = 7)		*p*-value	Progression due to pre-existing lesions (n = 33)		*p*-value
	High (n = 23)	Low (n = 17)		High (n = 4)	Low (n = 3)		High (n = 19)	Low (n = 14)	
Age at chemotherapy									
≤ 63	10 (43.5%)	8 (47.1%)		2 (50.0%)	2 (66.7%)		8 (42.1%)	6 (42.9%)	
> 63	13 (56.5%)	9 (52.9%)	0.822	2 (50.0%)	1 (33.3%)	0.629	11 (57.9%)	8 (57.1%)	0.966
Degree of progress									
Locally advanced	7 (30.4%)	4 (23.5%)		1 (25.0%)	1 (33.3%)		6 (31.6%)	3 (21.4%)	
Visceral metastases	16 (69.6%)	13 (76.5%)	0.454	3 (75.0%)	2 (66.7%)	0.714	13 (68.4%)	11 (78.6%)	0.405
Stage									
III or IV	11 (47.8%)	7 (41.2%)		0 (0.0%)	0 (0.0%)		11 (57.9%)	7 (50.0%)	
Rec	12 (52.2%)	10 (58.8%)	0.676	4 (100.0%)	3 (100.0%)	–	8 (42.1%)	7 (50.0%)	0.653
ER status									
Negative	13 (56.5%)	6 (35.3%)		2 (50.0%)	1 (33.3%)		11 (57.9%)	5 (35.7%)	
Positive	10 (43.5%)	11 (64.7%)	0.184	2 (50.0%)	2 (66.7%)	0.629	8 (42.1%)	9 (64.3%)	0.208
PgR status									
Negative	15 (65.2%)	8 (47.1%)		2 (50.0%)	1 (33.3%)		13 (68.4%)	7 (50.0%)	
Positive	8 (34.8%)	9 (52.9%)	0.251	2 (50.0%)	2 (66.7%)	0.629	6 (31.6%)	7 (50.0%)	0.284
HER2 status									
Negative	21 (91.3%)	16 (94.1%)		4 (100.0%)	3 (100.0%)		17 (89.5%)	13 (92.9%)	
Positive	2 (8.7%)	1 (5.9%)	0.615	0 (0.0%)	0 (0.0%)	–	2 (10.5%)	1 (7.1%)	0.616
Ki67									
Low	9 (39.1%)	11 (64.7%)		1 (25.0%)	1 (33.3%)		8 (42.1%)	10 (71.4%)	
High	14 (60.9%)	6 (35.3%)	0.110	3 (75.0%)	2 (66.7%)	0.714	11 (57.9%)	4 (28.6%)	0.093
Nuclear grade									
1, 2	11 (47.8%)	12 (70.6%)		2 (50.0%)	2 (66.7%)		9 (47.4%)	10 (71.4%)	
3	12 (52.2%)	5 (29.4%)	0.150	2 (50.0%)	1 (33.3%)	0.629	10 (52.6%)	4 (28.6%)	0.153
Objective response rate									
ORR	9 (39.1%)	7 (41.2%)		0 (0.0%)	1 (33.3%)		9 (47.4%)	6 (42.9%)	
Non-ORR	14 (60.9%)	10 (58.8%)	0.896	4 (100.0%)	2 (66.7%)	0.429	10 (52.6%)	8 (57.1%)	0.797

*ER* estrogen receptor, *PgR* progesterone receptor, *HER2* human epidermal growth factor receptor, *ORR* objective response rate

### Effects of TIL expression and differences in progression type upon prognosis

**Fig. 3:**
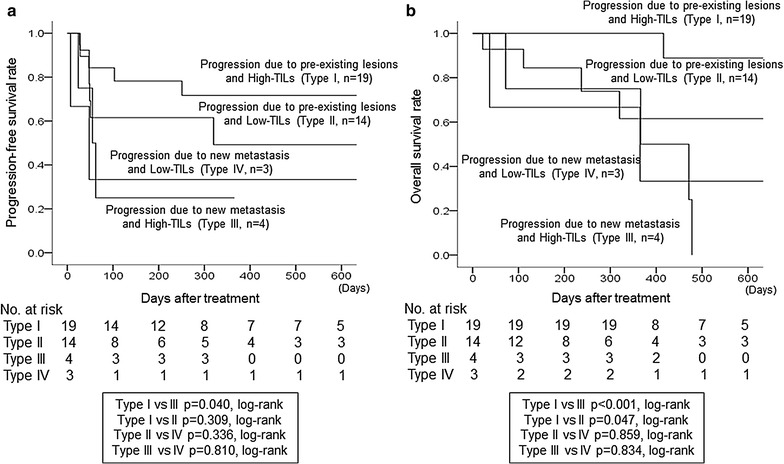
Effects of TIL expression and differences in progression type upon prognosis. In 19 cases, individuals with type I progression had significantly longer PFS compared to those with type III progression (p = 0.040, log-rank) (**a**). Furthermore, individuals with type I progression had significantly longer OS compared to those with type III and type II progression (p < 0.001 and p = 0.047, respectively; log-rank) (**b**)

**Fig. 4:**
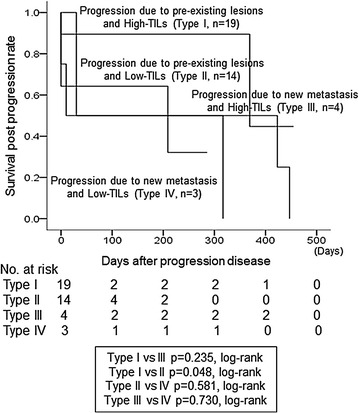
Survival post progression. Individuals with type I progression had significantly prolonged survival post progression (SPP) compared to those with type II progression (p = 0.048, log-rank)

**Fig. 5:**
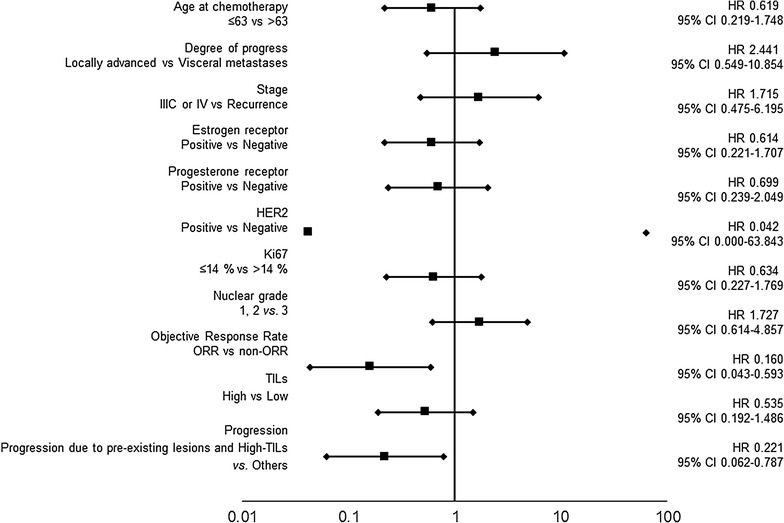
Forest plots. A univariate analysis that validate the effect of overall survival showed that “high objective response rate” and “progression due to pre-existing lesions and high-TILs” were considered as factors for a good prognosis (p = 0.006; HR = 0.160) (p = 0.020; HR = 0.221)

In 19 cases, individuals with type I progression had significantly longer PFS compared to those with type III progression (p = 0.040, log-rank) (Fig. 3). Furthermore, individuals with type I progression had significantly longer OS compared to those with type III and type II progression (p < 0.001 and p = 0.047, respectively; log-rank). Individuals with type I progression had significantly prolonged SPP compared to those with type II progression (p = 0.048, log-rank) (Fig. 4). A univariate analysis that validate the effect of OS showed that high ORR and type I progression were considered as factors for a good prognosis (p = 0.006; HR = 0.160) (p = 0.020; HR = 0.221) (Fig. 5). A multivariate analysis also showed that these were independent factors of good prognosis (p = 0.003; HR = 0.065) (p = 0.006; HR = 0.105) (Table 2).

**Table 2:** Univariate and multivariate analysis with respect to overall survival in 40 patients with eribulin chemotherapy for locally advanced or metastatic breast cancer

Parameters	Univariate analysis			Multivariate analysis		
	Hazard ratio	95% CI	*p*-value	Hazard ratio	95% CI	*p*-value
Age at chemotherapy						
≤ 63 vs. > 63	0.619	0.219–1.748	0.365			
Degree of progress						
Locally advanced vs. visceral metastases	2.441	0.549–10.854	0.241			
Stage						
IIIC or IV vs. recurrence	1.715	0.475–6.195	0.411			
ER						
Positive vs. negative	0.614	0.221–1.707	0.350			
PgR						
Positive vs. negative	0.699	0.239–2.049	0.514			
HER2						
Positive vs. negative	0.042	0.000–63.843	0.396			
Ki67						
≤ 14% vs. > 14%	0.634	0.227–1.769	0.384			
Nuclear grade						
1, 2 vs. 3	1.727	0.614–4.857	0.300			
Objective response rate						
ORR vs. non-ORR	0.160	0.043–0.593	0.006	0.065	0.011–0.388	0.003
TILs						
High vs. low	0.535	0.192–1.486	0.230			
Progression						
Progression due to pre-existing lesions and high-TILs vs. others	0.221	0.062–0.787	0.020	0.105	0.021–0.532	0.006

*ER* estrogen receptor, *PgR* progesterone receptor, *HER2* human epidermal growth factor receptor, *ORR* objective response rate, *TILs* tumor-infiltrating lymphocytes, *CI* confidence interval

## Discussion

Patients with MBC who underwent chemotherapy with eribulin in two international phase III clinical trials (Study 305 [eribulin monotherapy versus physician’s choice of treatment in patients with metastatic breast cancer, EMBRACE] and Study 301) had prolonged OS [11, 12]. Only pertuzumab [17–19] and trastuzumab emtansine (T-DM1) [20], other than eribulin, were proven to prolong the OS of individuals with HER2-positive breast cancer. The prolongation of OS due to chemotherapy is challenging in individuals with MBC because of the therapy’s relative biological mildness. However, other treatment options are also available. Although this therapy along with bevacizumab improved PFS, which has a higher response rate, it did not significantly affect OS (E2100, AVADO, RIBBON-1) [21–24]. Due to this reason, in addition to signal pathway blocking and cytocidal pharmacological actions, the TME is considered important in increasing OS in individuals with MBC who are on chemotherapy. The OS Kaplan–Meier curve in Studies 305 and 301 and the clinical evaluation of pertuzumab and trastuzumab (CLEOPATRA) trial showed a delayed separation curve during immunotherapy, suggesting that tumor immune response may be involved in these chemotherapy regimens [11, 12, 17, 18]. Thus, monitoring iTME through TILs is a key factor in predicting the therapeutic effect of chemotherapy with eribulin.

The TNM classification of tumor factor has conventionally been used as an indicator of cancer prognosis. However, differences in prognosis were found even when the degree of progression was the same. Therefore, the host factors of inflammatory response and nutritional status and TME monitoring as new indicators have been a topic of interest [7, 25–27]. That is, cancer progression is determined not only by the characteristics of the cancer cells themselves but also by the interactions between the cancer cells and TME, such as the epithelial–mesenchymal transition (EMT) and immune response [7, 28]. The effect of the immune responses in the TME of cancer host on prognosis and the prediction of the therapeutic effects of chemotherapy have also been reported [5–7, 29]. Based on basic research, eribulin has an inhibitory effect on the TME, including EMT suppression and tumor vascular remodeling [9, 10, 30]. Our previous study demonstrated that evaluating TILs before the start of eribulin therapy helped in the prediction of its therapeutic effect in individuals with triple-negative breast cancer [13]. Moreover, this study showed that patients with PPL who have good iTME conditions had a good prognosis.

In contrast, PD in the RECIST diagnostic criteria is classified into PPL and PNM, and individuals with PNM had a poorer prognosis than those with PPL in Studies 305 and 301 [3]. The difference between these two progression types is that PNM involves invasion into peripheral tissues and metastasis to other organs, which explains the course of poor prognosis, whereas the PPL does not involve metastasis but only invasion to peripheral tissues [2, 3].

The study has limitations since it involves a retrospective analysis of a small sample size. However, to the best of our knowledge, this study first investigated the progression types by evaluating the iTME in patients with MBC who undergoing chemotherapy, with an increased OS that was achieved through chemotherapy with eribulin. In the future, differences in progression types should also be considered in clinical practice to determine the best treatment options.

## Conclusions

In conclusion, this study showed that patients with PPL who have good iTME conditions had a good prognosis. In brief, the effects of eribulin chemotherapy suggested that patients with progressive-type breast cancer that proliferates in a good TME may have a good prognosis.

## Additional file

**Additional file 1: Figure S1.** Region of histopathological TIL evaluation. TILs were measured by examining the occupation ratio of immune cells present in the tumor stroma of hematoxylin and eosin stained specimens at ×400 magnification. Proportional scores of 3, 2, 1, and 0 were given if the area of stroma containing lymphoplasmacytic infiltration around invasive tumor cell nests comprised > 50% **(A)**, > 10–50% **(B)**, ≤ 10% **(C)**, and 0% **(D)**, respectively.

## Abbreviations

RECIST: response evaluation criteria in solid tumors
PPL: progression due to pre-existing lesions
PNM: progression due to new metastasis
PD: progressive disease
TILs: tumor infiltrating lymphocytes
iTME: immune tumor microenvironment
TME: tumor microenvironment
eribulin: eribulin mesylate
MBC: locally advanced or metastatic breast cancer
OS: overall survival
ORR: objective response rate
PFS: progression free survival
SPP: survival post progression
CR: complete response
PR: partial response
H.E.: hematoxylin and eosin
ER: estrogen receptor
PgR: progesterone receptor
HER2: human epidermal growth factor receptor 2
HR: hazard ratio
CI: confidence interval
EMBRACE: eribulin monotherapy versus physician’s choice of treatment in patients with metastatic breast cancer
T-DM1: trastuzumab emtansine
CLEOPATRA: clinical evaluation of pertuzumab and trastuzumab
EMT: epithelial–mesenchymal transition
