# Supplementary figures and images for: Study on the progression types of cancer in patients with breast cancer undergoing eribulin chemotherapy and tumor microenvironment
Source: J Transl Med. 2018 Mar 9;16:54. doi: 10.1186/s12967-018-1443-5 (PMC5845371; doi:10.1186/s12967-018-1443-5)

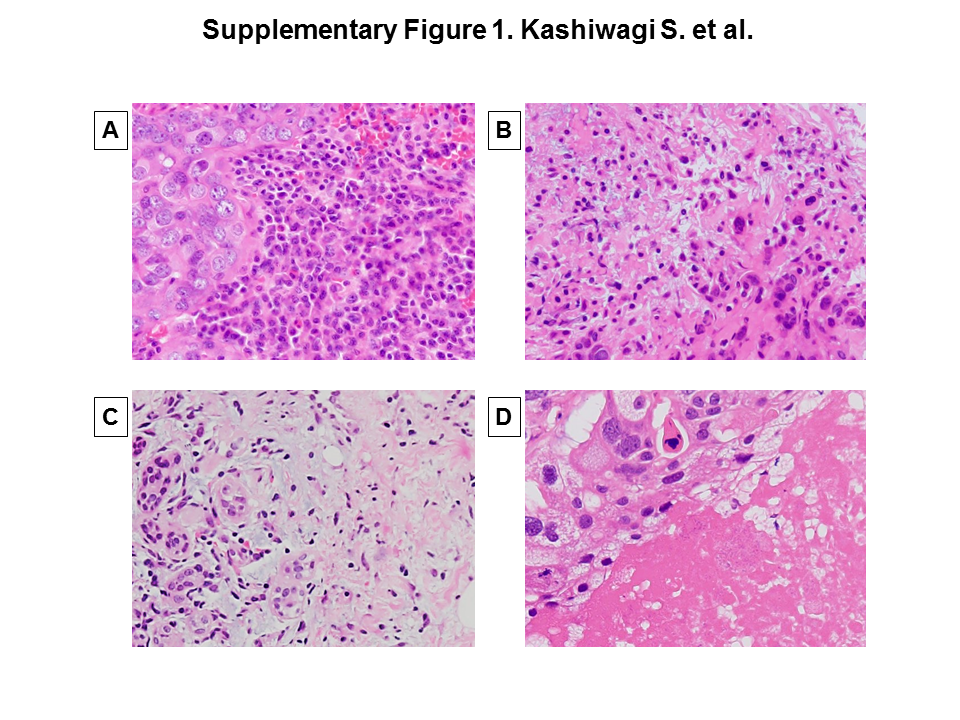

Supplement: Supplementary file 1 — Additional file 1: Figure S1. Region of histopathological TIL evaluation. TILs were measured by examining the occupation ratio of immune cells present in the tumor stroma of hematoxylin and eosin stained specimens at ×400 magnification. Proportional scores of 3, 2, 1, and 0 were given if the area of stroma containing lymphoplasmacytic infiltration around invasive tumor cell nests comprised > 50% (A), > 10–50% (B), ≤ 10% (C), and 0% (D), respectively. [file 12967_2018_1443_MOESM1_ESM.tif]
